# A Six-Tap iToF Imager with Wide Signal Intensity Range Using Linearization of Linear–Logarithmic Response

**DOI:** 10.3390/s25247551

**Published:** 2025-12-12

**Authors:** Tomohiro Okuyama, Haruya Sugimura, Gabriel Alcade, Seiya Ageishi, Hyeun Woo Kwen, De Xing Lioe, Kamel Mars, Keita Yasutomi, Keiichiro Kagawa, Shoji Kawahito

**Affiliations:** 1 Graduate School of Medical Photonics, Shizuoka University, Hamamatsu 432-8011, Japan; 2 Graduate School of Integrated Science and Technology, Shizuoka University, Hamamatsu 432-8011, Japan; 3 Graduate School of Science and Technology, Shizuoka University, Hamamatsu 432-8561, Japan; 4 Research Institute of Electronics, Shizuoka University, Hamamatsu 432-8011, Japan; 5 SUiCTE Co., Ltd., Hamamatsu 432-8011, Japan; 6 Shizuoka Institute of Science and Technology, Fukuroi 437-0032, Japan

**Keywords:** indirect time-of-flight (iToF), short-pulse, multi-tap, linearized linear–logarithmic response, dynamic range extension, retroreflective targets, pixel-wise correction

## Abstract

Time-of-flight (ToF) image sensors must operate across a wide span of reflected-light intensities, from weak diffuse reflections to extremely strong retroreflections. We present a signal-intensity range-extension technique that linearizes the linear–logarithmic (Lin–Log) pixel response for short-pulse multi-tap indirect ToF (iToF) sensors. Per-pixel two-region (2R) and three-region (3R) models covering the linear, transition, and logarithmic regimes are derived and used to recover a near-linear signal. Compared with a two-region approach that does not linearize the transition region, the 3R method substantially improves linearity near the knee point if extremely high linearity is required. Experiments with a six-tap iToF imager validate the approach. Depth imaging shows that linearization with common parameters reduces average error but leaves pixel-wise deviations, whereas pixel-wise 3R linearization yields accurate and stable results. Range measurements with a retroreflective target moved from 1.8–13.0 m in 0.20 m steps and achieved centimeter-level resolution and reduced the linearity-error bound from ±6.7%FS to ±1.5%FS. Residual periodic deviations are attributed to small pulse-width mismatches between the illumination and demodulation gates. These results demonstrate that Lin–Log pixels, combined with pixel-wise three-region linearization, enable robust ToF sensing over an extended dynamic range suitable for practical environments with large reflectance variations.

## 1. Introduction

Accurate distance measurement using time-of-flight (ToF) sensors has been actively studied and applied in diverse indoor and outdoor environments. Numerous techniques suppress background light [1,2,3,4,5,6,7], and some ensure robustness under challenging conditions such as fog [8]. Another critical factor is the intensity of the reflected light, which strongly depends on target reflectance [9,10,11,12,13,14]. In practical environments, targets typically have a diffusive surface, but sometimes they have very strong reflection. Retroreflective materials—widely used for road signs and nighttime vehicle visibility—can produce reflected signals 10–1000 stronger than those from white diffuse targets, depending on structure and measurement distance. To address these requirements, various dynamic-range-enhancing techniques have been developed. For example, multi-tap ToF sensors have been combined with adaptive exposure control, either per subframe or per time window, to optimize the number of accumulated pulses [4,5]. While these approaches work well when intensity scales are reflected with distance, they become less effective when multiple targets at similar distances exhibit large differences in reflectivity. Multi-tap more than 3 taps ToF sensors allocate multiple time windows within a single frame by incorporating several charge demodulators per pixel. Additionally, methods such as LOFIC (lateral overflow integration capacitor) [15,16,17] and sub-pixel [18,19] architectures enhance dynamic range. However, applying these techniques to multi-tap ToF pixels requires a relatively large pixel area because extra capacitors for extending dynamic range are necessary for every tap. Among dynamic range extension techniques, the use of pixel’s linear–logarithmic (Lin–Log) response [20,21,22,23,24] characteristics has a distinct advantage in smaller multi-tap pixels, because this technique does not need extra capacitors or circuits in pixel for extending dynamic range. However, the prior techniques directly using the Lin–Log characteristics are not suitable for ToF sensors, because the nonlinearity of the pixel response due to the linear–log characteristics causes a large nonlinearity in distance measurements.

In this paper, we investigate the feasibility of using a Lin–Log ToF sensor for accurate distance measurement via the use of algorithm-based linearization of the linear–log characteristics. This technique allows us to measure accurately the distance of objects with both weak and extremely strong reflections, such as those from retroreflective materials. We present a method to restore the linear signal from the linear–logarithmic response using a theoretical model, evaluate its accuracy through simulation, and validate its effectiveness via experiments with actual retroreflective targets.

## 2. Sensor Operation

### 2.1. Principle of Linear–Logarithmic (Lin–Log) Pixel Operation

**Figure 1:**
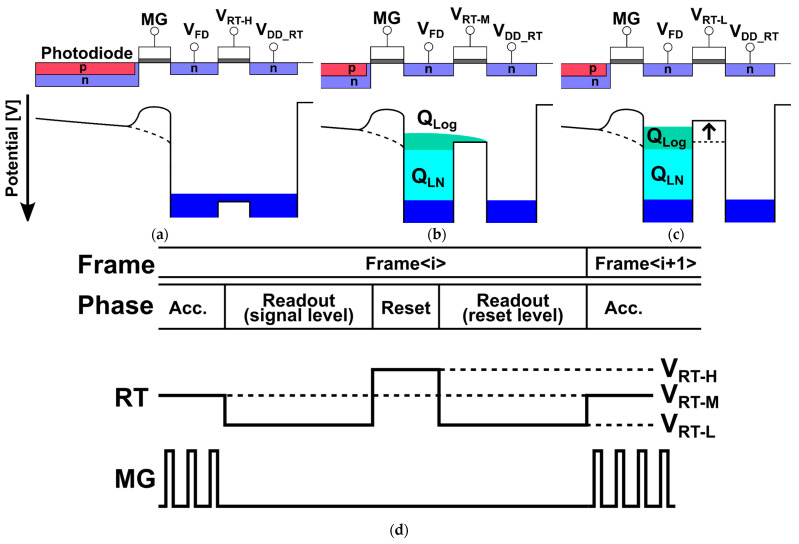
Concept of linear–logarithmic (Lin–Log) operation using three reset-gate levels. (**a**) Reset. The gate is $V_{RT-H}$. The floating diffusion (FD) is cleared to $V_{DD\_RT}$. (**b**) Accumulation. The gate is $V_{RT-M}$. A small leak to $V_{DD\_RT}$ appears. The response is linear at low signal (light-blue region) and becomes logarithmic near the knee (green region). (**c**) Readout. The gate is $V_{RT-L}$. The FD is isolated while the signal level is sampled. (**d**) Timing of $V_{RT-H}$, $V_{RT-M}$, and $V_{RT-L}$ during reset, accumulation, and readout.

Figure 1 shows how the proposed Lin–Log response is achieved by changing the biasing to the reset gate only. The reset supply node voltage is $V_{DD\_RT}$. The three reset-gate levels are $V_{RT-H}$, $V_{RT-M}$, and $V_{RT-L}$. In the reset phase (Figure 1a), the gate is set to $V_{RT-H}$, which fully resets the floating diffusion (FD) to $V_{DD\_RT}$. During accumulation (Figure 1b), the gate is held at $V_{RT-M}$; the reset transistor then operates in subthreshold and provides a controlled leakage path to $V_{DD\_RT}$ when the signal light intensity is very large. As a result, the FD response is linear at low signal and smoothly transitions to a logarithmic region when the FD potential becomes low enough that the $V_{RT-M}$ leakage limits further increase, which is called the knee point, as shown by $Q_{LN}$ and $Q_{Log}$ in Figure 1b. For readout, the gate is switched to $V_{RT-L}$ to turn the reset transistor off and isolate the FD while the signal level is sampled. To prevent the FD from receiving another signal and background light charges during readouts, the modulation gate (MG) and draining gate (DG) are set to low and high, respectively. Figure 1d shows the pixel phases and the corresponding RT levels ($V_{RT-H}$, $V_{RT-M}$ and $V_{RT-L}$). Readout uses two sub-phases: signal-level sampling at $V_{RT-L}$, then a short reset at $V_{RT-H}$ followed by reset-level sampling at $V_{RT-L}$. This pixel uses FD nodes for charge storage, providing a large full-well capacity, but the kTC noise remains in the readout noise. Therefore, in the sensor presented in this work, the distance resolution (range precision) is mainly limited by kTC noise. Although this sensor does not employ true CDS, we expect that ToF pixels with charge storage for true CDS operation could also adopt a Lin–Log response and linearization scheme based on the method described in this paper.

### 2.2. Lin–Log Operation for Six-Tap SP iToF Sensor

**Figure 2:**
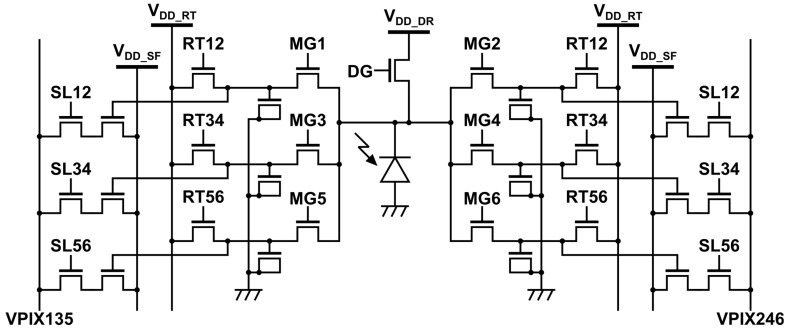
Six-tap SP iToF pixel circuit for Lin–Log operation. Two analog column readout lines (VPIX135 and VPIX246) carry the tap voltages. Tap selection uses SL12, SL34, and SL56. Pair-wise reset uses RT12, RT34, and RT56 referenced to the reset supply $V_{DD\_RT}$.

Figure 2 shows the pixel circuit. The two analog column readout lines (VPIX135 and VPIX246) carry the tap voltages. Three tap-select clocks (SL12, SL34, and SL56) connect one tap pair at a time to the corresponding column lines. Three reset clocks (RT12, RT34, and RT56) drive the reset transistors for the same pair and, during reset, connect the FDs to the reset supply $V_{DD\_RT}$.

**Figure 3:**
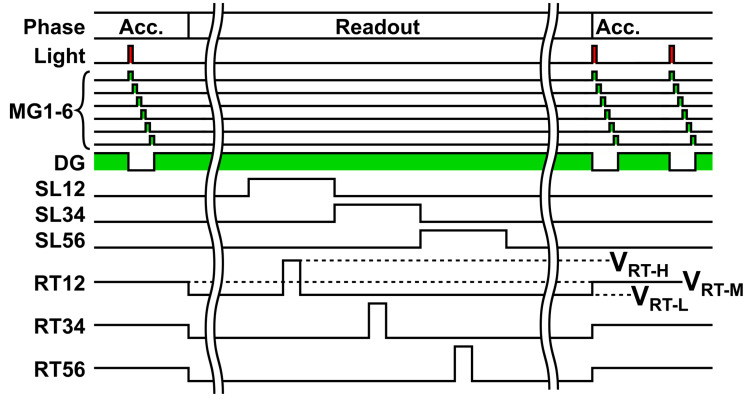
Timing of control signals for global exposure. Accumulation uses $V_{RT-M}$. For each tap pair—1 and 2, then 3 and 4, then 5 and 6—the sequence is: signal-level sampling at $V_{RT-L}$, a short reset at $V_{RT-H}$ to $V_{DD\_RT}$, then reset-level sampling at $V_{RT-L}$.

Figure 3 shows the control sequence for the global exposure of the Lin–Log response 6-tap pixel. In one modulation cycle, the light source emits short pulses and the demodulator transfers charge to each tap. The modulation cycle sequentially applying modulation gates, MG1, MG2, …, MG6 and draining gate DG is repeated during accumulation. The number of modulation cycles, typically 1000 to 100,000, is determined by the required signal intensity. During accumulation, all reset clocks (RT12, RT34, and RT56) remain at $V_{RT-M}$, which causes the FDs to exhibit the Lin–Log behavior described in Figure 1. At the start of readout, the light source turns off, and all reset clocks transition to $V_{RT-L}$. This prevents charge loss from the FDs. During readout, each tap pair is processed in turn: 1 and 2, then 3 and 4, then 5 and 6. The selected pair is connected to the column readout lines. The signal level is sampled while $V_{RT-L}$ is held. A short pulse at $V_{RT-H}$ resets the FDs to the reset supply $V_{DD\_RT}$. The reset level is then sampled at $V_{RT-L}$. The same steps repeat for the next pair.

## 3. Theory of Linear–Logarithmic Response Restoration

### 3.1. Derivation of FD Voltage

**Figure 4:**
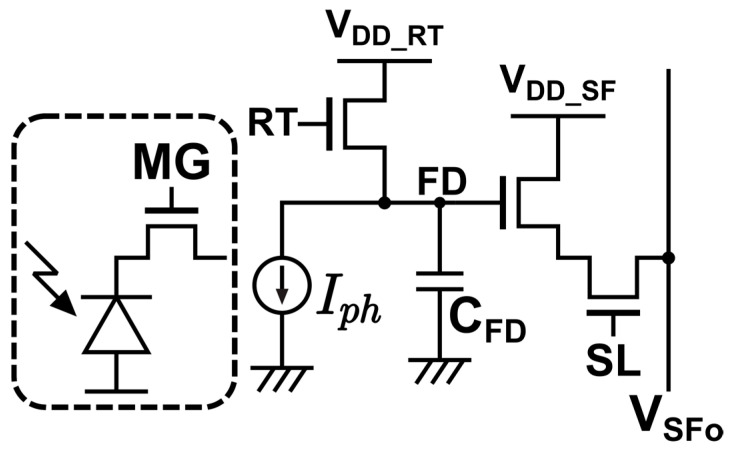
Circuit model to derive theoretical linear–logarithmic response of the pixel.

Figure 4 shows the circuit model to derive the theoretical linear–logarithmic response of the pixel.

For simplicity, the photodiode and charge demodulator MG are modeled as a constant current source $I_{ph}$. An equation for the response of the floating diffusion (FD) voltage $V_{FD}$ for the circuit including the subthreshold current $I_{sub}$ of the reset transistor and the capacitance at the FD $C_{FD}$ is given by:

(1) $$I_{ph}=-C_{FD}\frac{dV_{FD}}{dt}+I_{sub}.$$

Using the reset level of the gate voltage $V_{RT}$, and the threshold voltage $V_{TH}$ of the reset transistor, the subthreshold current is expressed as

(2) $$I_{sub}=I_{s0}\exp\left(\frac{V_{RT}-V_{FD}-V_{TH}}{nV_{t}}\right),$$

where $V_{t}$ (=*kT*/*q*) is the thermal voltage and n is a factor determined by the ratio of the depletion capacitance and gate capacitance, which is known to be greater than 1 due to the depletion capacitance appearing in series with the oxide capacitance [25]. Here, $I_{s0}$ denotes the drain current of the reset transistor when $V_{RT}-V_{FD}=V_{TH}$. This condition corresponds to the boundary between the subthreshold and strong-inversion regions.

The FD voltage after the accumulation of photo charge at the FD with the accumulation time $T_{a}$ is derived by solving this differential equation using an initial condition that $I_{sub}\left(t=0\right)=I_{sub0}$ as

(3) $$V_{FD}=V_{RT}-V_{TH}-nV_{t}\ln\left[\;\frac{I_{ph}}{I_{sub0}}\cdot \frac{1}{\;1+\left(\frac{I_{ph}}{I_{sub0}}-1\right)exp\left(-\frac{T_{a}I_{ph}}{nV_{t}C_{FD}}\right)}\;\right].$$

There is another way of solving the differential equation using an initial condition that $I_{sub}\left(t=0\right)=0$. However, this condition requires the linear–logarithmic response to be written in a piecewise form that depends on the photocurrent level, which makes the overall expression more complicated. Using the condition of $I_{sub}\left(t=0\right)=I_{sub0}$, which is extremely small but not exactly zero, the solution can be written in a concise and refined form. From the same reason, the FD voltage change due to the accumulation of photo current, ${∆V}_{FD}$ as a function of $I_{ph}$ is defined as ${∆V}_{FD}=V_{FD}\left(I_{ph}\right)-V_{FD}\left(I_{sub0}\right).$ Then $\Delta V_{FD}$ is expressed as

(4) $$\Delta V_{FD}=nV_{t}\ln\left[\;\frac{\frac{I_{ph}}{I_{sub0}}}{\;1+\left(\frac{I_{ph}}{I_{sub0}}-1\right)exp\left(-\frac{T_{A}I_{ph}}{nV_{t}C_{FD}}\right)}\;\right].$$

The derived expression for $\Delta V_{FD}$ shows that the pixel response is linear for small signals and logarithmic for large signals. To illustrate this dual behavior, useful approximations can be given under both limiting conditions.

- Low-input condition $\left(I_{ph}\exp\left(-T_{A}I_{ph}/\left(nV_{t}C_{FD}\right)\right)\gg I_{sub0}\right)$:

(5) $$\Delta V_{FD}=\frac{T_{A}}{C_{FD}}\;I_{ph}.$$

In this region, the FD voltage drop is governed by the linear integration response of the capacitance.

- High-input condition ($I_{ph}\exp\left(-T_{A}I_{ph}/\left(nV_{t}C_{FD}\right)\right)\ll I_{sub0}$:

(6) $$\Delta V_{FD}\;=\;nV_{t}\ln\frac{I_{ph}}{I_{sub0}}.$$

In this region, the FD voltage responds logarithmically to the photo current and Equation (6) is valid under the assumption that $I_{ph}\gg I_{sub0}$. In Equation (6), the term $-nV_{t}\ln I_{sub0}$ acts as an offset caused by the subthreshold leakage.

### 3.2. Reconstruction of Linear Signals from Log-Compressed Output

To derive a formula to reconstruct the virtually linearized signal from the linear–logarithmic response curve, the following definition for the virtually linearized signal voltage at the FD node is introduced as

(7) $$V_{L}\equiv \frac{T_{A}}{C_{FD}}\;I_{ph}.$$

To refine the formula, a coefficient to express the slope factor of the logarithmic region is also defined as

(8) $$A\equiv nV_{t}.$$

A voltage level corresponding to the knee point in the linear–logarithmic response, $V_{K}$ is introduced and it is defined as

(9) $$V_{K}=\frac{T_{A}}{C_{FD}}I_{ph,K}=A\ln\frac{I_{ph,K}}{I_{sub0}}.$$

Here, $I_{ph,K}$ denotes the photocurrent corresponding to the knee point $V_{K}$. Hence,

(10) $$\frac{I_{ph}}{I_{sub0}}\;=\;\frac{V_{L}}{V_{K}}\exp\frac{V_{K}}{A}.$$

Substituting this relation into Equation (4) gives $∆V_{FD}$ as

(11) $${∆V}_{FD}\;=\;V_{K}+A\ln\left[\frac{V_{L}}{V_{K}+V_{L}\exp\left(\frac{V_{K}-V_{L}}{A}\right)-V_{K}\exp\left(\frac{-V_{L}}{A}\right)}\right].$$

The output signal swing at the source follower output $V_{out}$ is assumed to be exactly equal to $∆V_{FD}$, i.e., the source follow gain is unity, the relationship between $V_{L}$ and $V_{out}$ is expressed as

(12) $$V_{L}\;=\;V_{K}\exp\frac{V_{out}-V_{K}}{A}+\exp\frac{V_{out}-V_{L}}{A}\left(V_{L}-V_{K}\exp\frac{-V_{K}}{A}\right).$$

In Equation (12), the term $V_{K}exp$($-V_{K}/A)$ is negligibly small because this term is calculated to be approximately $3.3\times 10^{-15}$ V for a typical $V_{K}$ and A of 1 V and 30 mV, respectively. Then a concise and refined formula to express the relationship between $V_{L}$ and $V_{out}$, using only the two parameters $V_{K}$ and A, is obtained as

(13) $$V_{L}\;=\;V_{K}\exp\frac{V_{out}-V_{K}}{A}+V_{L}\exp\frac{V_{out}-V_{L}}{A}.$$

This is the basic equation to reconstruct a linear signal from the output with linear–logarithmic characteristics. Based on Equation (13) and by solving $V_{L}$ for a given $V_{out}$ and constants of $V_{K}$ and A, the linear signal is reconstructed. However, as Equation (13) is an implicit function including exponential functions, solving Equation (13) in real time requires high computational capabilities in implemented ToF camera systems. To simplify the reconstruction process, two types of approximation for solving Equation (13) are presented here.

1. Two-region (2R) Method

In this method, the response can be approximated in two limiting cases:

(14) $$V_{L}=\left\{\begin{array}{cc} \;V_{out},\;\;\;\;\;\;\;\;\;\;\;\;\;\;\;\;\;\;\;\;\;\;\;\; & V_{out}\leq V_{K}\\ \;V_{K}\exp\frac{V_{out}-V_{K}}{A}, & V_{out}{>V}_{K} \end{array}\right.$$

This approximation is accurate for the regions of $V_{out}-V_{K}>5A$ (logarithmic) or $V_{out}-V_{K}<-5A$ (linear), because the second term and first term, respectively, in the right-hand side of Equation (13), are negligible. However, for the region of ${-5A<V}_{out}-V_{K}<5A$ (transitional region connecting the two), particularly for the region of $V_{out}-V_{K}\approx 0$, the error of Equation (14) from Equation (13) is relatively large.

2. Three Region (3R) Method

In the three region (3R) method, the linearity of $V_{L}$ is improved by using another function in the transitional region connecting the linear and logarithmic region. If $\left|V_{L}-V_{out}\right|\ll A$, the following approximation holds:

(15) $$\exp\frac{V_{out}-V_{L}}{A}\approx 1+\frac{V_{out}-V_{L}}{A}.$$

Using Equation (15), Equation (13) is approximated as

(16) $$V_{L}\approx V_{out}+A\frac{V_{K}}{V_{L}}\exp\frac{V_{out}-V_{K}}{A}.$$

Since $V_{L}$ of the right-hand side is a slowly changing function in the transitional region, the following equation, obtained by assuming $V_{L}$ of the right-hand side is constant, will give a good model to describe the transitional region.

(17) $$V_{L}\;=\;V_{out}+G_{t}\exp n_{T}\frac{V_{out}-V_{K}}{A},$$

where $G_{t}$ and $n_{t}$ are constants to adjust the linearity of the transitional region. Then the 3R method is expressed as

(18) $$V_{L}=\left\{\begin{array}{cc} \;V_{out},\;\;\;\;\;\;\;\;\;\;\;\;\;\;\;\;\;\;\;\;\;\;\;\;\;\;\;\;\;\;\;\;\;\;\;\;\;\;\;\;\;\;\;\;\;\;\;\;\;\;\;\;\;\;\;\;\;\;\; & V_{out}<V_{T1}\;\;\;\;\;\;\;\;\;\;\;\;\\ \;V_{out}+G_{t}\;\exp\left(n_{T}\;\frac{V_{out}-V_{K}}{A}\right),\;\;\;\;\;\;\;\;\;\;\; & V_{T1}<V_{out}<V_{T2}\\ \;V_{K}\exp\frac{V_{out}-V_{K}}{A},\;\;\;\;\;\;\;\;\;\;\;\;\;\;\;\;\;\;\;\;\;\;\;\;\;\;\;\;\;\;\;\;\;\;\; & V_{T2}<V_{out}\;\;\;\;\;\;\;\;\;\;\;\; \end{array}\right.$$

where $V_{T1}$ and $V_{T2}$ are the threshold to switch between linear and transitional, and between transitional and logarithmic, respectively.

From an implementation point of view, the linearization is not computationally heavy. Each pixel value is corrected by a piecewise function based on the equations above (for example, Equations (14) and (18)). In the logarithmic region, the correction uses a few basic arithmetic operations and one evaluation of an exponential function per pixel. The computational load is therefore similar to standard tone-curve or gamma correction in an image signal processor and is suitable for real-time ToF processing. When pixel-wise parameters are used, additional memory is needed to store them for each pixel. In this work, we apply pixel-wise correction and store two parameters, $V_{K}$ and A, per pixel. Such pixel-wise calibration data are already common in ToF cameras (e.g., for per-pixel offset and gain correction), so the required memory and processing for the proposed linearization are comparable to those of conventional ToF systems.

## 4. Simulation Results

### 4.1. Linear–Logarithmic Response by Circuit Simulation

**Figure 5:**
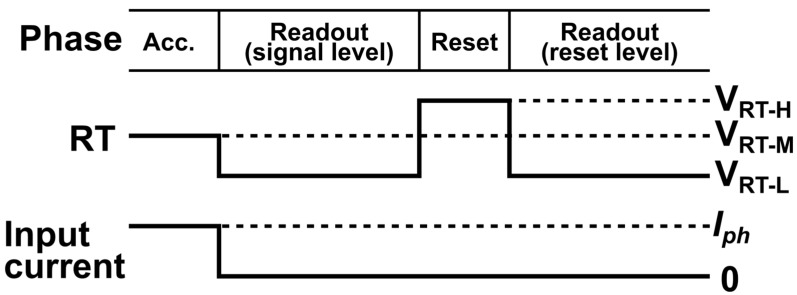
Timing diagram used in the circuit simulations, showing the operation phases, the reset-gate signal RT at $V_{RT-L}$, $V_{RT-M}$, $V_{RT-H}$, and the input current $I_{ph}$ during accumulation and readout.

Circuit simulations were conducted based on the equivalent circuit shown in Figure 4. The timing of the photocurrent $I_{ph}$ and the reset-gate signal RT is shown in Figure 5. During the accumulation period, the photocurrent $I_{ph}$ was modeled as a constant current and the reset-gate signal RT was biased at $V_{RT-M}$. During the readout period, $I_{ph}$ was set to zero and RT was fixed at 0 V. The main simulation parameters were as follows:

- accumulation time $T_{A}=1.5$ ms
- FD capacitance $C_{FD}=2.0$ fF
- photocurrent $I_{ph}$ ranged from 0 to 10 pA

**Figure 6:**
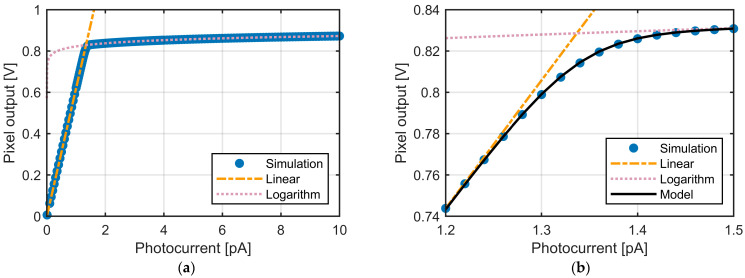
Simulation results of the pixel output as a function of photocurrent. (**a**) Comparison of the simulated output with the linear–logarithmic response function and its linear-region and logarithmic region approximations over the full photocurrent range (0–10 pA). (**b**) Enlarged view of the transition region (1.2–1.5 pA) illustrating the detailed agreement between the simulation, the analytical model, and the approximations.

The difference between the reset voltage and the signal voltage of the pixel output $V_{SFo}$ was defined as the final output signal. The resulting output voltages were compared with the linear–logarithmic response function derived in Section 3, as well as with its linear- and logarithmic-region approximations, as shown in Figure 6.

It can be observed that the simulation results fit very well with either the linear-region approximation or the logarithmic-region approximation over the respective ranges. Around the intersection of the two approximations, the simulated outputs are smoothly connected between the linear and logarithmic behaviors. Furthermore, the Lin–Log response model derived from the equivalent circuit shows excellent agreement with the simulation results.

**Figure 7:**
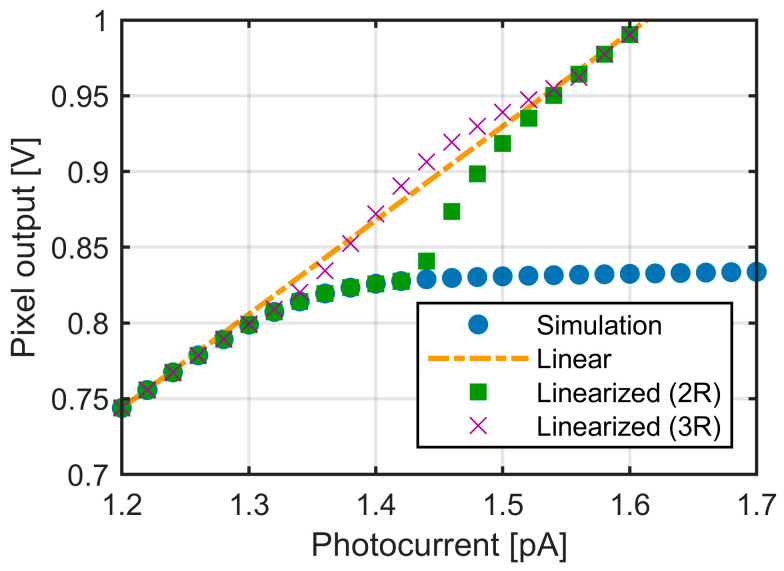
Linearization of the simulated pixel output to restore the linear signal. The results are shown for two approaches: two-region (2R) linearization using $V_{K}$ as the boundary, and three-region (3R) linearization using thresholds $V_{T1}$ and $V_{T2}$.

To recover the linear signal from the pixel output exhibiting a linear–logarithmic response, linearization was applied to the circuit simulation results. Two methods were examined: (i) Two-region (2R) method described by Equation (14) and (ii) Three-region (3R) method described by Equation (18). The results of both methods are plotted in Figure 7.

While the 2R method shows good agreement in both the low- and high-signal regions, the error is concentrated around the boundary point. The 3R method alleviates this issue and improves the linearity in the transition region. When the two-region method (2R) was applied, the restored signal around $V_{K}$ deviated from the ideal linear response by up to −52 mV, while the three-region method (3R) significantly improved the linearity near $V_{K}$, reducing the deviation to within ±15 mV. The 3R method requires only one additional threshold and substantially improves the accuracy in the vicinity of $V_{K}$. The practical impact of this improvement will be further discussed in the subsequent ToF simulation results.

### 4.2. iToF Range Measurement Simulation with Linear–Logarithmic Response

Using the pixel characteristics obtained from the circuit simulations, time-of-flight (ToF) range measurement simulations were performed for a multi-tap SP iToF sensor employing linear–logarithmic response pixels. The assumed operating conditions for the simulations are summarized in Table 1.

Based on the modeling of iToF range measurements under a perfectly diffuse surface reported in [26], the model was extended to the case of retroreflective targets. In this simulation, the retroreflector was assumed to return approximately 30 times stronger reflected light compared with a white diffuse board, and the reflectance parameter was therefore set to 3000%. The average optical power of the light source was set to 600 mW, which is a common specification for widely used iToF cameras [1]. With a light pulse cycle of 300 ns and a pulse width of 10 ns, the corresponding peak power becomes 18 W, maintaining the average optical power at 600 mW.

**Figure 8:**
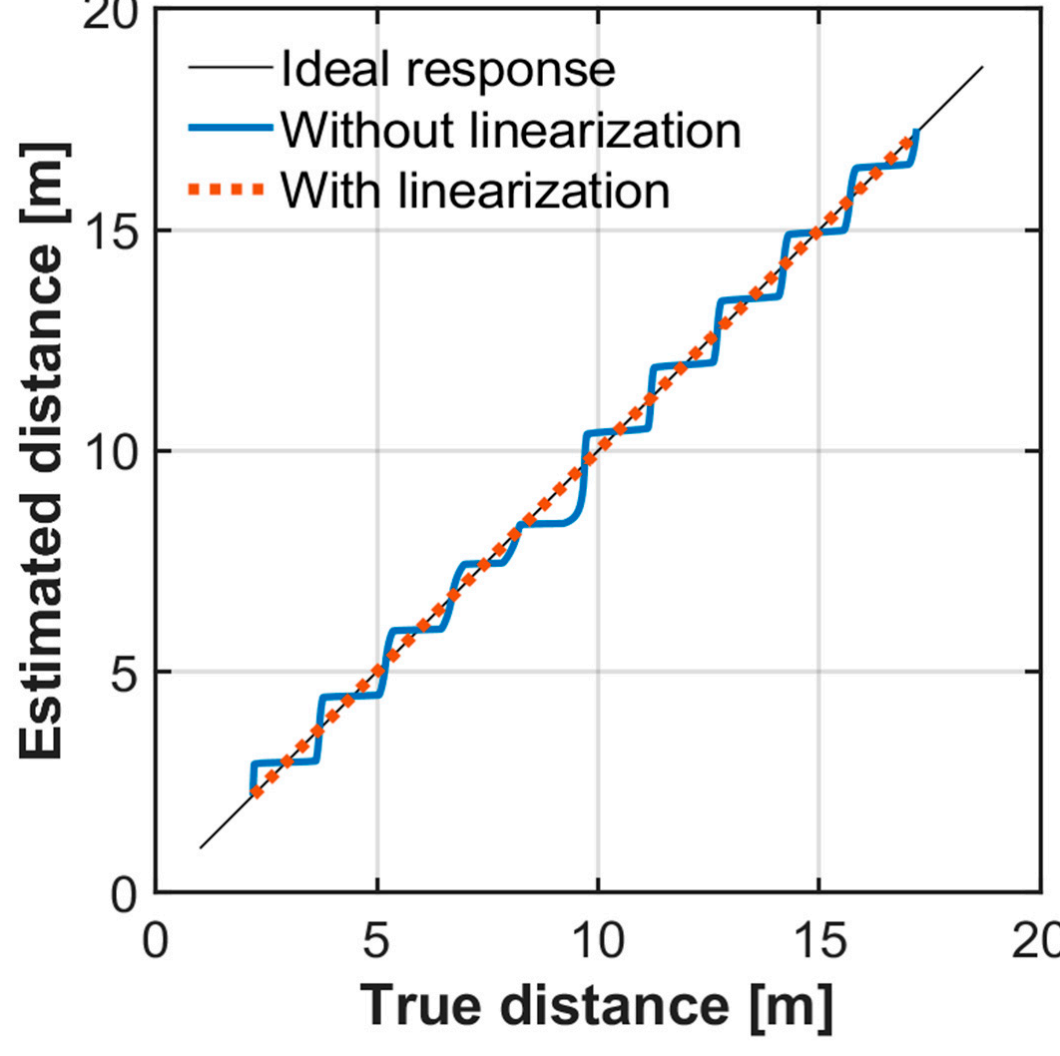
Simulated ToF range measurement results. With correction, the estimated distance exhibits a linear response, whereas without correction it shows a stepwise characteristic.

Figure 8 shows the simulated range measurement results showing the relationship between the true distance and the estimated distance. The blue solid line represents the result obtained without correcting the linear–logarithmic pixel response, while the red dashed line shows the result obtained after restoring the linear signal. With correction, the estimated distance exhibits a linear and accurate response with respect to the true distance. In contrast, without correction, the estimated distance displays a staircase-like characteristic, indicating significant nonlinearity errors occur.

In the hybrid-type iToF method [26,27,28] used here, the six taps capture the reflected light pulse in different time windows. When the width of the light pulse is equal to the width of a time window, the reflected pulse is distributed over two adjacent taps. The coarse distance is determined by finding the tap with the largest output, and the fine distance is calculated from the outputs of the taps in which the reflected pulse is detected. Under the simulation conditions of Figure 8, the reflected signal is so strong that, over the simulated distance range, the outputs of these taps operate in the logarithmic region of the Lin–Log response. In this region, the tap outputs change only slightly with the incident signal. As a result, the fine distance cannot be calculated accurately from the raw pixel outputs, and the estimated distance becomes stepwise without linearization. Therefore, when the tap outputs are in the logarithmic region, linearization of the Lin–Log response is essential for accurate distance estimation.

**Figure 9:**
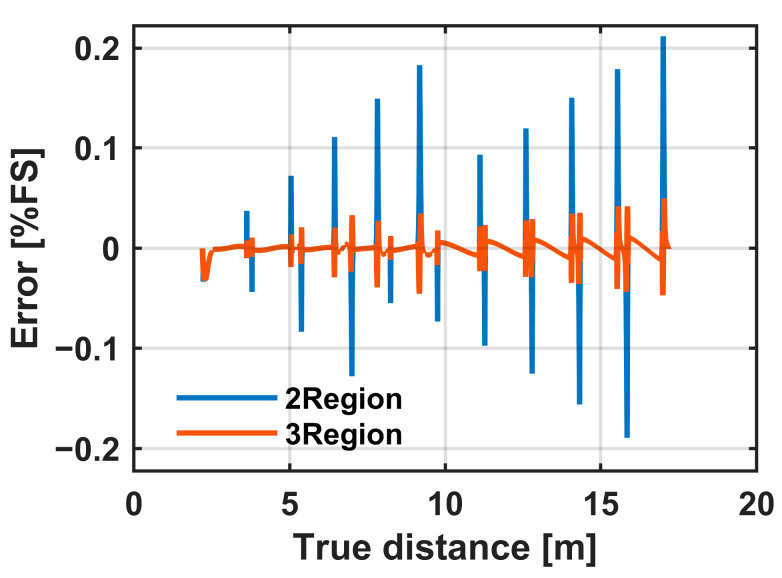
Comparison of linearity errors between the two-region and three-region linearization methods. Three-region method reduces errors to ±0.05%FS compared with ±0.22%FS for the two-region method, corresponding to a 76.4% reduction.

Figure 9 compares the linearity errors obtained using the 2R and 3R linearization methods. In both cases, the error becomes larger when the pixel output is around the knee point ($V_{K}$). However, the 3R method significantly reduces the error compared with the 2R method. The error bound for the two-region method was ±0.22%FS, while that for the three-region method was ±0.05%FS, corresponding to an improvement of a 76.4% reduction.

## 5. Experimental Results

### 5.1. Experimental Setup for iToF Camera Measurements

Range measurements were performed with a six-tap iToF image sensor [29]. The sensor architecture and on-chip readout are summarized in Table 2. The device was fabricated in a 0.11 μm CIS BSI process and comprises 1080 (H) × 488 (V) pixels with an 8.4 μm × 8.4 μm pitch; the chip size is 13.32 mm × 10.48 mm, and the line readout time is 6.018 ms. Column conversion uses a 12-bit FI/cyclic ADC with a correlated multiple sampling (CMS) gain of 2 [30] and a programmable-gain amplifier (PGA) gain of 0.8.

The operating conditions used for the range measurements are listed in Table 3. A Diamond Grade retroreflector served as the target. The optical system was set to F-number 1.4. Per measurement, the sensor accumulated 35,000 illumination pulses. The light source was driven with a 300 ns pulse cycle and a 15 ns pulse width (5.0% duty), and the system frame rate was 60 fps. These settings were chosen to reflect practical high-reflectivity operation.

To perform the linearization of the linear–logarithmic response and the correction for the linearity of the pixel-to-pixel variation, the parameters of knee point $V_{K}$ and logarithmic slope A included in the correction function were measured. A retroreflective target was placed at a distance of 2.0 m, and the trigger signal for controlling the light source was adjusted using a delay controller to emulate different time-of-flight (ToF) conditions. By measuring the pixel output response to the input signal, two points in the linear region and two points in the logarithmic region were extracted. From these four points, the parameters $V_{K}$ and A were determined for each pixel, and subsequently used for depth image reconstruction.

The linearization method applied here is based on the 3R (three-region) approach, as also discussed in the simulation results. The threshold voltages defining the three regions are determined from the parameters $V_{K}$ and A, optimized for each pixel.

### 5.2. Pixel Responses to Retroreflective Targets and Linearization

**Figure 10:**
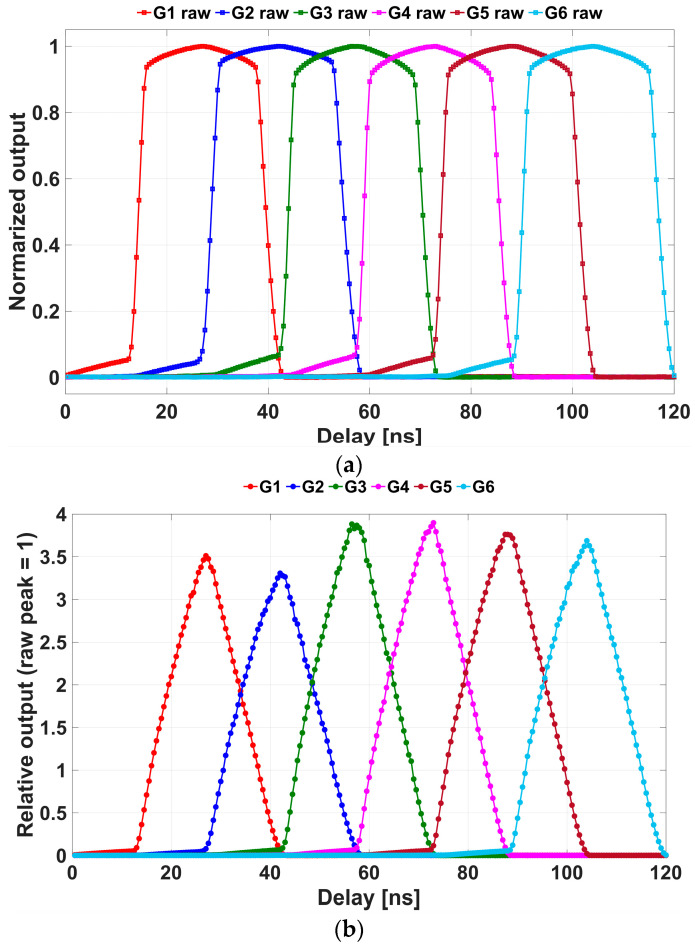
Measured pixel responses as functions of delay time. (**a**) Raw outputs of the six demodulation gates (G1–G6), normalized to their respective peaks. (**b**) Linearized outputs. These plots illustrate the method used to extract the linearization parameters $V_{k}$ and A.

Figure 10 shows the delay-sweep measurement used to derive per-pixel linearization parameters. A retroreflective target was placed at a distance of 2.0 m. The trigger driving the light source was shifted by a delay controller relative to the camera timing to emulate different ToF while keeping the setup unchanged. Figure 10a shows the raw outputs of the six demodulation gates (G1–G6) from a representative pixel, averaged across 200 frames and normalized to their respective peaks. These delay-dependent responses are then used to estimate, on a pixel-by-pixel basis, the parameters required for linearization (the knee level $V_{K}$ and the logarithmic-slope factor A). In this study, we used the same pulsed 940 nm laser as in the ToF measurements to estimate $V_{K}$ and A. From the standpoint of the Lin–Log pixel model, we expect that using the same light source is not strictly required if the calibration source provides a suitable intensity range under comparable conditions.

After estimating $V_{K}$ and A for each pixel, the linearization is applied to recover a near-linear response. Figure 10b shows the linearized outputs. The linearized curves exhibit the expected triangular profile and a higher relative amplitude, consistent with the inversion of log compression.

**Figure 11:**
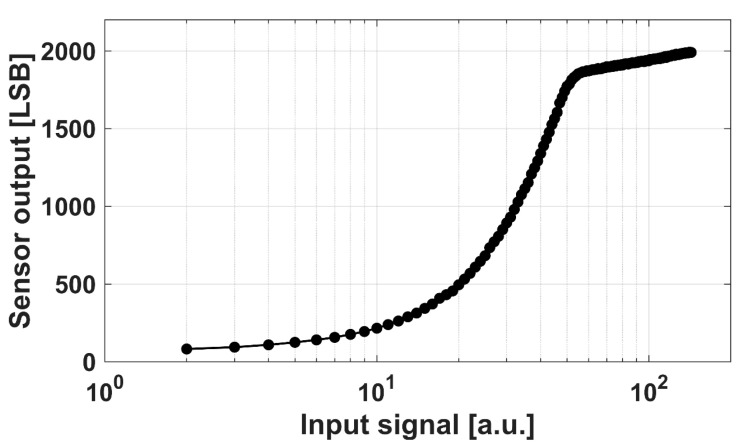
Measured Lin–Log photo-response of tap G3. Sensor output versus input signal level on a logarithmic horizontal axis. The curve shows a linear region at low input, followed by a knee and a logarithmically compressed region at high input signal.

To illustrate the Lin–Log photo-response more directly, Figure 11 shows the measured output of tap G3 versus the input signal level. The input level is defined from the delay settings used in the same measurement setup as Figure 10. The horizontal axis is logarithmic. The curve is approximately linear at low input levels and then shows a knee and a logarithmically compressed region at high input levels.

**Figure 12:**
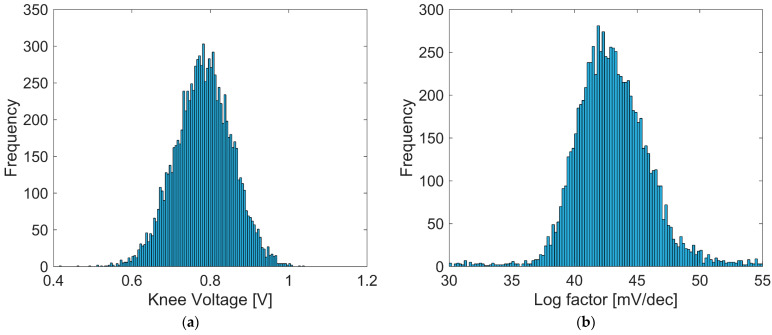
Histograms of the estimated Lin–Log parameters for tap G6 over 100 × 100 pixels of the array: (**a**) knee voltage $V_{K}$, (**b**) logarithmic factor A.

Figure 12 shows histograms representing the distributions of the measured parameters $V_{K}$ and A for tap G6 obtained from 100 × 100 pixels. The logarithmic slope factor is shown as $A\ln\left(10\right)$ in units of mV/dec. Similar distributions are observed for the other taps. The measured median value of the logarithmic slope factor is slightly smaller than the theoretical value for n = 1. This difference is considered to be due to the photocurrent being applied as repeated pulses generated by the pulsed light source and the demodulation gates, rather than as a constant photocurrent.

Prior to range calculation, the Lin–Log pixel outputs are linearized on a per-pixel basis using a three-region linearization whose thresholds are derived from $V_{K}$ and A. The same linearization procedure is applied both for the fixed-distance experiment in this section and for the varying-distance targets described in the following sections. After linearization, a 3 × 3 median filter is applied to the images to suppress isolated outliers while preserving edges, and the resulting images are then used for range computation.

### 5.3. Range Imaging of Retroreflector and Diffuse Targets

**Figure 13:**
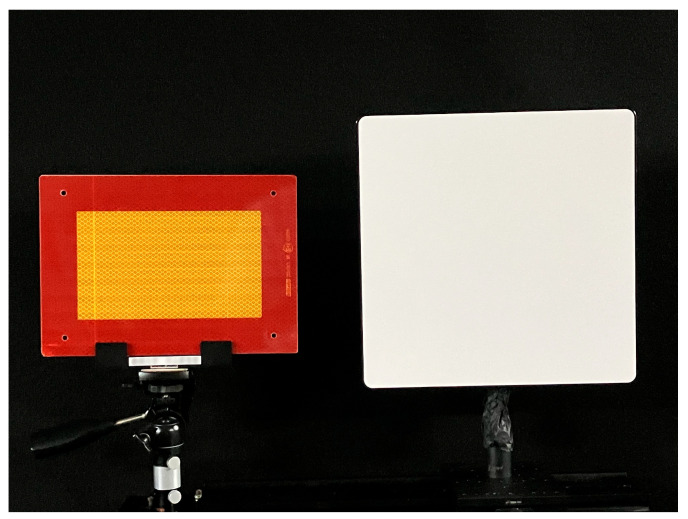
Experimental setup showing the two types of targets placed at the same position: a retroreflective plate (**left**) and a white diffuse reflector with a nominal reflectance value of 99% (**right**).

Using the linearization of the Lin–Log pixel outputs of the 6-tap iToF image sensor based on the 3R method given by Equation (18), range images were captured under the experimental setup shown in Figure 13, where two different targets were placed at the same position: a retroreflective plate and a white diffuse reflector with a nominal reflectance value of 99%. This measurement aims to compare the imaging characteristics of retroreflective and diffuse surfaces under identical conditions. For each pixel, the distance value is obtained by averaging 30 consecutive frames.

**Figure 14:**
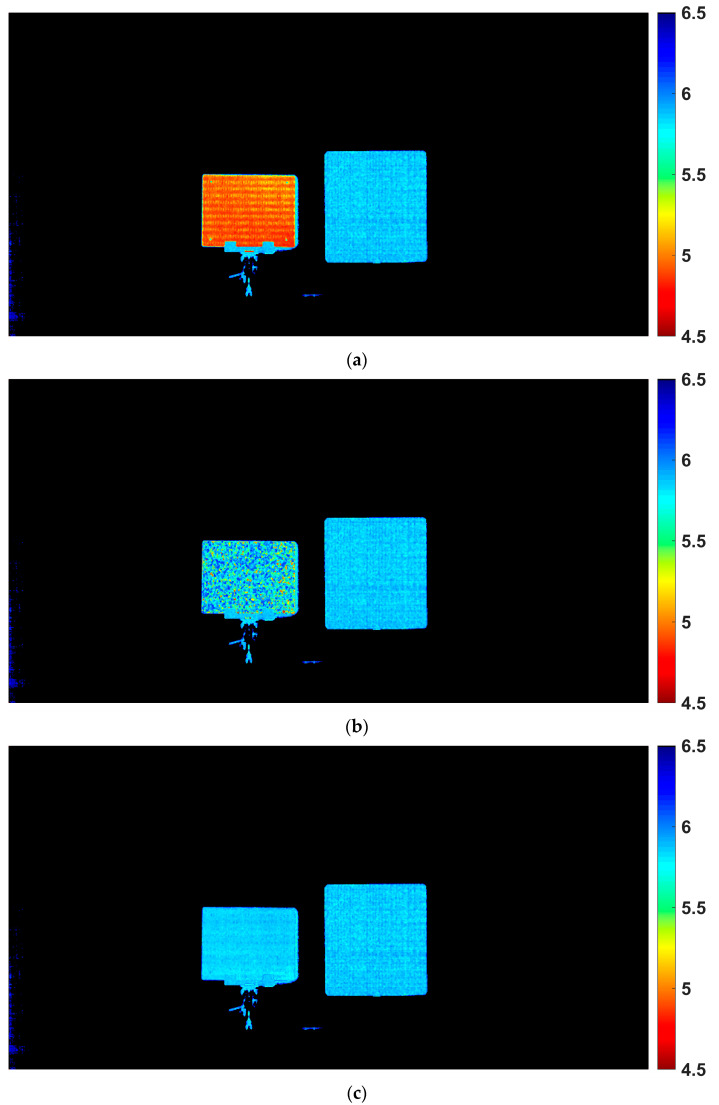
Measured depth images of the retroreflective (left) and diffuse (right) targets. (**a**) Without correction. (**b**) With global correction using common parameters. (**c**) With pixel-wise correction using individually optimized parameters.

Figure 14 shows the measured depth images of the retroreflective and diffuse targets placed side by side. Since the same raw data were used for all three cases, the diffuse target (right-hand side in Figure 14) naturally produced consistent results regardless of whether correction was applied. This is because the reflected signal is relatively weak and the tap outputs remain below the knee level $V_{K}$, where the pixel response is linear. Its median depth was 5.855 m with a standard deviation (STD) of 0.044 m across all conditions.

In the case without any correction of the linear–logarithmic response, as shown in Figure 14a, the retroreflective target (left-hand side in Figure 14) suffered from severe distortion due to the nonlinearity and incorrect depth estimation, showing a median depth of 4.949 m with an STD of 0.073 m. The depth difference between the retroreflective and diffuse targets was −0.907 m, indicating a large error for the retroreflective surface. When a global correction was applied using common parameters $V_{K}$ and A for all pixels in Figure 14b, the retroreflective target improved to a median depth of 5.831 m. The depth difference relative to the diffuse target was reduced to −0.024 m. The average values of $V_{K}$ and A across pixels were used as the common parameters. However, a large STD of the nonlinearity of 0.254 m remains, indicating large pixel-wise deviation. With pixel-wise correction, where individually optimized parameters $V_{K}$ and A were applied to each pixel, the retroreflective target achieved the most accurate reconstruction as shown in Figure 14c. The median depth was 5.846 m with a reduced STD of 0.028 m, and the difference relative to the diffuse target decreased further to −0.010 m. These results confirm that pixel-wise correction is essential for achieving accurate and stable depth measurements of retroreflective surfaces.

**Figure 15:**
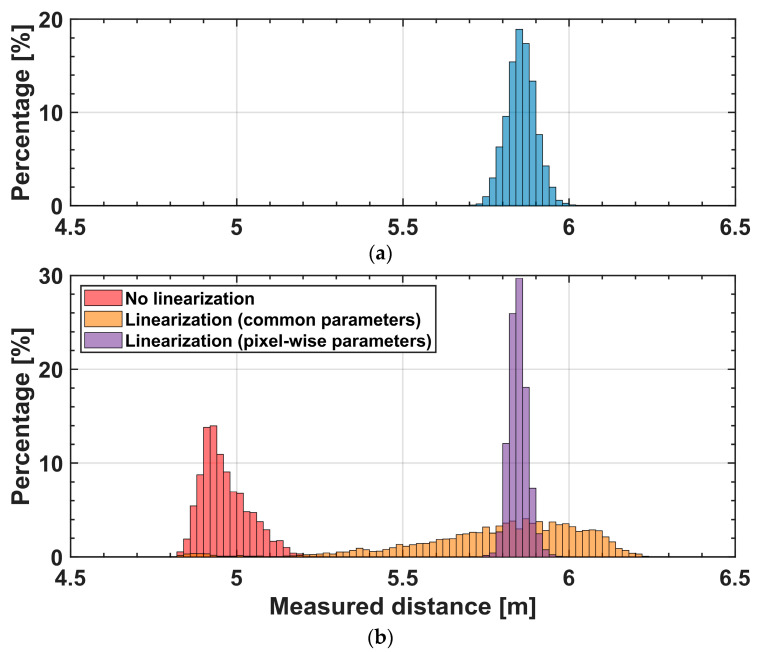
Histograms of measured distances for (**a**) a diffuse whiteboard and (**b**) a retroreflective target. The retroreflective results are shown for three cases: no linearization, linearization with common parameters, and linearization with pixel-wise parameters. Linearization with pixel-wise parameters produces the tightest and most accurate distribution.

Figure 15 shows histograms of the measured distance for the diffuse whiteboard and the retroreflective target using the same depth data as in Figure 14. Each histogram is computed from the distance values of a region of 100 × 50 pixels on the planar target. The whiteboard shows a narrow distribution around the correct distance. Because the diffuse whiteboard returns a much smaller light signal than the retroreflective plate, its SNR is lower, and the histogram width is strongly influenced by the readout noise. In contrast, the retroreflective target without linearization exhibits a broad and biased distribution. Applying a global correction with common parameters $V_{K}$ and A improves the median depth but still results in a wide spread of values due to pixel-wise inconsistencies. With pixel-wise linearization, the distance distribution shows a smaller spread around the correct value. As discussed around Equation (9), the parameter $V_{K}$ is mainly determined by the FD capacitance, that is, by the conversion gain of each pixel. Therefore, the use of per-pixel parameters reduces pixel-to-pixel variations in $V_{K}$ (and A), so that the spread of the distance values for the retroreflective plate becomes comparable to, or even smaller than, that of the diffuse target.

**Figure 16:**
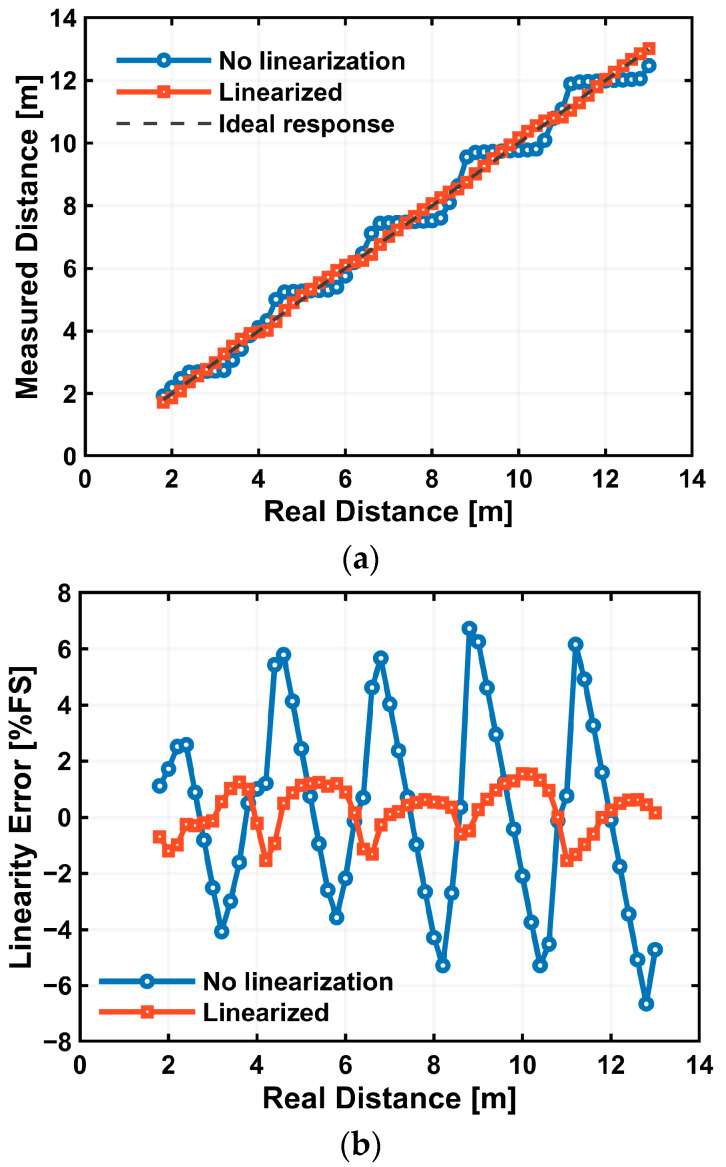
Range measurements with a retroreflective target (1.8–13.0 m, 0.20 m steps). (**a**) Measured versus true distance for raw Lin–Log output and linearized output; dashed line: ideal response. (**b**) Linearity error (%FS; FS = 11.2 m). Error bound improves from ±6.7%FS (No linearization) to ±1.5%FS (linearized).

Range measurements were performed by moving the retroreflective target from 1.8–13.0 m in 0.20 m increments. For each distance, the measured distance is taken as the mean over 10 × 10 pixels centered on the target. Figure 16a plots the measured distance versus the true distance for two cases, without linearization and with linearization. Without correction, the measured distance exhibits a staircase behavior characteristic of multi-tap iToF when the pixel output is compressed near the knee. With the proposed linearized Lin–Log response, the measurements closely follow a linear relationship with the true distance across the full range. Figure 16b shows the corresponding linearity error expressed in percent of full scale (%FS), where the full-scale range is 11.2 m. The case without linearization exhibits an error bound of ±6.7%FS, whereas the linearized case reduces the bound to ±1.5%FS—an improvement of approximately 4.5×. Residual periodic deviations are consistent with small mismatches between the illumination pulse width and the demodulation-gate pulse width; these can be mitigated by further optimization of the driving conditions.

**Figure 17:**
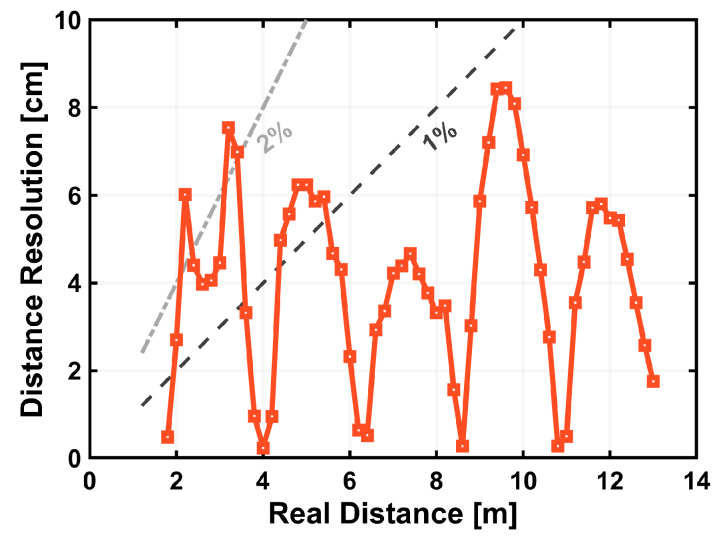
Distance resolution for the linearized Lin–Log output. Dashed lines indicate 1% and 2% of distance for reference.

Figure 17 shows the distance resolution obtained after restoring the linear signal from the Lin–Log pixel output. The resolution remains within a few centimeters over the entire range, corresponding to better than 1% relative accuracy, except at specific distances where the modulation introduces periodic variations. This modulation is consistent with a small mismatch between the illumination pulse width and the demodulation-gate pulse width and can be mitigated by further optimization of the driving conditions.

## 6. Discussion

### 6.1. Linearity, Resolution, and Periodic Deviations

Restoring the linear signal from the Lin–Log pixel output is essential for stable multi-tap iToF ranging for realizing very wide signal intensity range. With pixel-wise three-region linearization, the estimated distance follows the true distance closely (Figure 8 and Figure 16), whereas nonlinearized outputs exhibit pronounced stepwise artifacts. The distance resolution remains at the centimeter level across the measured range; however, periodic degradation appears at specific distances. These components are consistent with a slight mismatch between the illumination pulse width and the demodulation-gate pulse width (and/or their timing/shape). In practice, tighter timing alignment and pulse-width matching can mitigate this behavior without modifying the pixel architecture.

### 6.2. Reflection Model Gap: Simulation vs. Measurement

In the simulations, the retroreflective return is modeled as 30-fold that of a 100% diffuse surface at the same position. In the measurements, the retroreflective plate may produce an optical return approximately 1000 times stronger than the co-located diffuse target. Despite this much larger-than-assumed reflectance, accurate ranging was achieved by combining Lin–Log pixels with reconstruction of the linear signal, underscoring robustness under extremely high dynamic range.

### 6.3. Linearization Strategy: Two-Region vs. Three-Region; Global vs. Pixel-Wise

Circuit simulations indicate that the three-region (3R) strategy reduces error near the knee point compared with the two-region (2R) approach (Figure 9). In the measurements, however, the 3R method does not significantly improve linearity compared with the 2R method, because the nonlinearity discussed in the measurement section is at percent level. Since the 3R method is effective in achieving very high linearity when the required nonlinearity error is below 0.1%FS, its advantage is expected to be more pronounced in future developments of very accurate and highly robust iToF imagers. Notably, the three-region (3R) method requires only one additional threshold relative to the two-region (2R) scheme, so the computational overhead is small while the linearity gain around $V_{K}$ is substantial. Regarding the method of linearization, global linearization using common $V_{K}$ and A reduces gross bias but leaves pixel-level deviations (Figure 14). Pixel-wise linearization suppresses these deviations and yields uniform depth, enabling accurate reconstruction of retroreflective targets.

### 6.4. Assumptions, Limitations, and Future Work

The reflectance model was extended from diffuse to retroreflective targets; scenes with mixed materials and multipath interference were not analyzed here. Noise sources (shot, read, timing jitter) are reflected implicitly in the measured statistics; a unified model spanning linear and logarithmic domains would clarify how inverse Lin–Log mapping reshapes noise and SNR versus range and reflectance. Temperature variation and aging may shift $V_{K}$ and A. However, the temperature characteristics of these parameters can be incorporated by extending the Lin–Log response model, enabling compensation with modest measurement and computational overhead. Future work includes closing the loop on timing using the actual illumination and gate pulses to suppress the observed periodic deviations and streamlining fast in-field linearization flows for $V_{K}$ and A. In addition, a comprehensive statistical characterization over the complete pixel array, including distance-error distributions under various operating conditions, is left for future work.

## 7. Conclusions

This study has presented a dynamic-range extension technique for short-pulse multi-tap ToF image sensors based on the linearization of linear–logarithmic response pixels. A theoretical model for restoring the linear signal was established and validated through circuit simulations, highlighting the superior performance of the three-region linearization method over the simpler two-region approach. ToF simulations revealed that without proper linearization, multi-tap range estimation produces staircase-like errors, while linearized outputs yield accurate linear responses. Experimental results with an actual iToF camera further confirmed that pixel-wise linearization of $V_{K}$ and A is indispensable for accurate depth reconstruction. Histograms and depth images demonstrated that global linearization can mitigate gross errors but fails to suppress pixel-level deviations, whereas pixel-wise linearization ensures uniformity and precision. Ranging tests up to 13 m validated that the system achieves centimeter-level resolution, with periodic deviations explained by pulse width mismatches, suggesting further improvements through sensor drive optimization. Overall, the proposed Lin–Log response with pixel-wise linearization provides a practical and effective solution for extending the dynamic range of ToF sensors, enabling reliable operation in environments with both highly reflective and low-reflectance objects.

## Figures and Tables

**Table 1:** Simulation conditions assumed for multi-tap SP iToF range measurement using linear–logarithmic response pixels.

Parameter	Value
Reflectance of a perfectly diffuse surface	3000% (White: 95%)
Transmittance of optics (inc. lens and BPF)	80%
Lens F number	1.4
Quantum efficiency	20%
Number of demodulation gates	6
Area of photodiode	8.4 μm × 8.4 μm
Average optical power of light source	600 mW
Optical peak power of light pulse signal	18 W
Number of accumulation light pulse	$5000\;@\;\mathrm{SF}1\;(T_{A}=1.5\left[ms\right]$)$20,000\;@\;\mathrm{SF}2\;(T_{A}=6.0\left[ms\right]$)
Light pulse Cycle	300 ns
Pulse width	10 ns
Duty ratio	3.3%
FD capacitance	2.0 fF

**Table 2:** Specifications of the six-tap SP iToF sensor.

Parameter	Value
Process technology	0.11 μm CIS BSI
Number of pixels	1080(H) × 488(V)
Pixel size	8.4 μm × 8.4 μm
Number of taps	6 + 1 (drain)
Chip size	13.32 mm × 10.48 mm
ADC	12 bits FI/cyclic
CMS gain	2
PGA gain	0.8
Readout time	6.018 ms

**Table 3:** Experimental conditions for the iToF range measurements.

Parameter	Value
Reflector grade	Diamond Grade™
Lens F number	1.4
Light pulse Cycle	300 ns
Pulse width	15 ns
Duty ratio	5.0%

## Data Availability

The original contributions presented in this study are included in the article. Further inquiries can be directed to the corresponding author.
